# Prophage Activation: An In Silico Platform for Identifying Prophage Regulatory Elements to Inform Phage Engineering Against Drug-Resistant Bacteria

**DOI:** 10.3390/life15091417

**Published:** 2025-09-08

**Authors:** Saher Musrrat, Zequan Han, Kai Wang, Yunhai Huang, Yanhui Xiang, Sen Liu, Wen Yin

**Affiliations:** 1 State Key Laboratory of Quantitative Synthetic Biology, Shenzhen Institute of Synthetic Biology, Shenzhen Institutes of Advanced Technology, Chinese Academy of Sciences, Shenzhen 518055, China; saher@siat.ac.cn; 2 University of Chinese Academy of Sciences, Beijing 100049, China; 3 Cooperative Innovation Center of Industrial Fermentation (Ministry of Education & Hubei Province), Key Laboratory of Fermentation Engineering (Ministry of Education), Hubei University of Technology, Wuhan 430068, China; 1025387313hzq@gmail.com; 4 State Key Laboratory of Bioreactor Engineering and School of Biotechnology, East China University of Science and Technology, Shanghai 200237, China; karlking4736@gmail.com (K.W.); yunian314@163.com (Y.H.)

**Keywords:** lysogenic–lytic switch, bacteriophage engineering, regulatory element editing, multidrug-resistant bacteria, phage therapy

## Abstract

Multidrug-resistant bacterial infections pose a severe global health threat, highlighting the urgent need for innovative therapeutic options beyond traditional antibiotics. Phage therapy, which employs bacteriophages to infect and eradicate pathogenic bacteria, specifically offers a promising solution. However, the lack of well-characterized therapeutic phages has limited their broader clinical use. A critical aspect of activating the lytic potential of dormant prophages involves the strategic manipulation of transcription factor binding sites (TFBS), which function as pivotal regulatory nodes governing the transition between lysogenic dormancy and lytic activation. Our platform utilizes advanced bioinformatics tools to accurately identify and analyze TFBS, facilitating the targeted redesign or replacement of these sites to disrupt host-mediated repression. By systematically simulating modifications of these regulatory ‘switches,’ our platform computationally predicts reduced repressor activity, suggesting the potential for prophage activation and bacterial cell lysis. This novel methodology not only broadens the spectrum of therapeutic bacteriophages but also establishes a basis for individualized phage-based therapies, presenting a robust strategy to address the escalating challenge of antibiotic-resistant infections. By enabling the precise identification and engineering of TFBS, our platform signifies a transformative advancement in phage biology, effectively bridging the divide between computational analysis and therapeutic application.

## 1. Introduction

Antimicrobial resistance (AMR) occurs when certain microorganisms, particularly bacteria, develop the ability to withstand the effects of antibiotics and other therapeutic agents [1]. The World Health Organization has identified AMR as a leading global health priority [2,3,4]. Phage therapy, which entails administering virulent bacteriophages to patients with clinical infections to swiftly eradicate pathogenic bacteria, presents a promising strategy for combating drug-resistant infections [5,6,7,8]. In this process, prophages already present in the host can protect the bacterium from reinfection by related phages through several mechanisms, including modification of host surface receptors, prevention of DNA injection, and repression of early phage genes [9]. In the case of temperate phages such as lambda, this process involves the prophage’s master repressor protein. This repressor, which is continuously expressed by the prophage to sustain its lysogenic state, can bind to transcription factor binding sites (TFBS) on the DNA of an incoming, related phage [10,11]. This binding action suppresses the expression of early lytic genes necessary for the replication of the superinfecting phage. As a result, phage therapy that demonstrated efficacy in vitro may not succeed in vivo due to the latent immunity conferred by resident prophages. Identifying an effective phage for a particular infection is challenging, as many phages are strain-specific. However, some broad-host-range phages have been reported that can infect across multiple species or even genera [12,13]. Consequently, new phages often need to be isolated for each infection, which delays the treatment process and complicates the broader therapeutic application [14,15].

To overcome this challenge, numerous bioinformatics tools have been developed to identify prophages within bacterial genomes: PhiSpy [16], Prophage Hunter [17], DBSCAN [18], PHATEST [19], Seeker [20], and Phage Finder [21]. For instance, Prophage Hunter uses a scoring system based on sequence similarity searches against phage protein databases and machine learning classification using their genetic features to estimate the probability that a prophage is active. However, predicting whether a prophage is truly capable of induction and replication in situ remains challenging, as its activity can be influenced by host factors and specific environmental triggers that are not easily captured by genomic analysis alone [22,23]. Tools like PropagAtE [24] aim to infer activity from metagenomic read coverage data, identifying the prophage region. They may annotate a repressor gene but do not predict which specific DNA sequences the repressor will bind to on a potentially unrelated therapeutic phage. Therefore, it is difficult to engineer therapeutic phages that can avoid repression and successfully replicate within lysogenized target cells with the current prophage platform.

Prophage Activation Platform pipeline is complementary to existing tools, providing additional regulatory insights (TFBS, promoter analysis) that other prophage tools do not currently offer. A key component of this approach is a scoring framework that quantitatively assesses the impact of sequence alterations within TFBS. The pipeline begins with prophage region identification and attachment site localization through genome sequence upload. This is followed by promoter region detection, then analysis and modification of TFBS using an integrated scoring system that minimizes repressor binding while preserving promoter activity. The final stage enables genome sequence editing based on an optimized regulatory change. This AI-driven platform offers a streamlined and modular approach to therapeutic phage design. This framework is specifically designed to decrease repressor binding affinity while maintaining promoter activity. Notably, Prophage Activation represents the first online AI tool for the design of therapeutic phages aimed at evading host bacterial SIE. The novelty of this research is that it takes a fresh approach by focusing on TFBS within dormant prophages instead of looking for new phages out in the natural world. The Prophage Activation platform thus provides significant new opportunities for addressing drug-resistant bacterial strains and developing next-generation antibacterial therapies.

## 2. Materials and Methods

### 2.1. Data Collection

Complete bacterial genome sequences were retrieved from the National Center for Biotechnology Information (NCBI) genome database (https://www.ncbi.nlm.nih.gov/, accessed 5 June 2023), and bacteriophage genome sequences were obtained from the Actinobacteriophage database (https://phagesdb.org/, accessed on 12 July 2023) [25]. These meticulously curated sequences (Supplementary Table S1) constituted the primary training dataset for our deep learning model, facilitating the model’s ability to discern distinguishing features between host genomic regions and prophage sequences. The combined dataset ensured comprehensive coverage of prophage diversity across various bacterial species, forming the foundation for model development within the Prophage Activation framework perspective on the interactions between multidrug-resistant bacterial hosts and their prophages.

### 2.2. Pipeline Analysis of Prophage Activation

Software can be reached via https://github.com/Lou-lab-01/Prophage_Activation, (accessed on 5 May 2025). To identify and activate prophages within bacterial genomes, we developed a comprehensive multi-step computational pipeline that integrates established bioinformatics tools with custom Python scripts and machine learning models. First, bacterial genomes in FASTA format were annotated for prophage detection using Prokka version 1.14.6 [26] to predict protein-coding sequences and tRNAs. The resulting annotated GenBank files were further refined by re-annotating tRNA sites using ARAGORN version 1.2.41 [27]. Subsequently, protein sequences were then queried against a locally constructed UniProt TrEMBL viral protein database using DIAMOND BLASTP version 2.0.15 [28]. An E-value threshold of ≤10^−7^ was applied to ensure a balance between sensitivity (detecting true prophage-related hits) and specificity (reducing false positives). This cutoff was selected based on its reliability in previous prophage detection studies. For very distant homologs, more stringent cutoffs or alternative methods (e.g., HMM-based approaches) may provide additional sensitivity. Accordingly, this step was describled in detail to clarify its application specifically to prophage and att site detection. These genes were mapped back to genomic coordinates and clustered into candidate prophage regions using a custom Python script, with clusters defined as containing at least six phage-like genes within intergenic distances of ranging from 3000 to 10,000 base pairs. To delineate prophage boundaries, attL and attR sites were predicted using a motif-based approach that considered conserved flanking sequences.

Promoter regions were identified using a hybrid scoring methodology that integrated position weight matrix (PWM) scores with a Random Forest (RF) classifier. The RF model was trained on a dataset of 500 curated bacterial promoter sequences from RegulonDB (v10.5) [29] and 500 randomly selected intergenic regions from the *Escherichia coli* K-12 MG1655 reference genome (NCBI RefSeq: NC_000913.3). Input features included PWM scores, k-mer frequencies (3-mers and 4-mers), and GC content. The final RF model, consisting of 100 trees with a maximum depth of 10, achieved an accuracy of 93.5%, a sensitivity of 91.2%, and a Matthews correlation coefficient (MCC) of 0.89.

Following promoter detection, TFBS were identified using a two-step motif-based approach. First, candidate motifs were located via sliding-window searches against PWMs derived from bacterial promoter consensus sequences and curated motif databases (RegulonDB v10.5), applying a cutoff of E ≤ 10^−7^ to retain statistically significant hits. Candidate motifs were filtered according to genomic context, with priority given to sites upstream of prophage repressors or in proximity to promoter regions. To complement this, de novo motif discovery was performed using MEME (v5.5.3), and motifs were annotated against known databases with TOMTOM, enabling the detection of both conserved and novel TFBS.

Finally, in the repressor interaction simulation, repressor genes identified within prophage regions were computationally excised to simulate prophage induction. This was followed by confirming the disruption of TFBS repressor interactions, alongside the verification of intact downstream lytic modules. The successful identification of these modules indicated a transition from lysogeny to lysis, thereby effectively simulating prophage activation within the host genome.

## 3. Results

### 3.1. The Prophage Activation Platform: Proof of Concept

Our Prophage Activation platform is conceptualized as an extensive and multifaceted computational framework, meticulously crafted to systematically explore the complex realm of integrated prophages across diverse bacterial genomes. At its foundation, the platform’s design philosophy emphasizes precision, universality, and modulatory control to bridge the gap between genomic discovery and therapeutic intervention.

The operational framework initiates with high-throughput genomic analysis, wherein users upload complete bacterial genome sequences, regardless of bacterial lineage. These sequences are then parsed and processed through a custom-built pipeline, developed in Python utilizing Biopython and other bioinformatics libraries. The platform conducts a comprehensive genome-wide scan, identifying key prophage signatures such as integrase genes, repressor genes, terminase subunits, capsid and tail protein-encoding regions, as well as flanking sequence features, including direct repeats and variations in GC content. These features are integrated with intergenic distance metrics and local gene density to delineate prophage boundaries with high precision. The analysis is automated and optimized for server-side performance, facilitating rapid processing and consistent detection across a broad spectrum of bacterial genomes. To enhance accuracy further, the system incorporates reference phage protein databases for the functional validation of candidate regions and employs quality filters to reduce false positives. This methodology enables the platform to reliably detect both intact and cryptic prophages across diverse bacterial hosts. This initial identification serves as a foundation for more in-depth, targeted investigations. For example, when processing the *Mycobacterium smegmatis* strain mc^2^ 155 genome (NC_008596.1), the platform successfully identified six distinct prophage regions.

**Figure 1:**
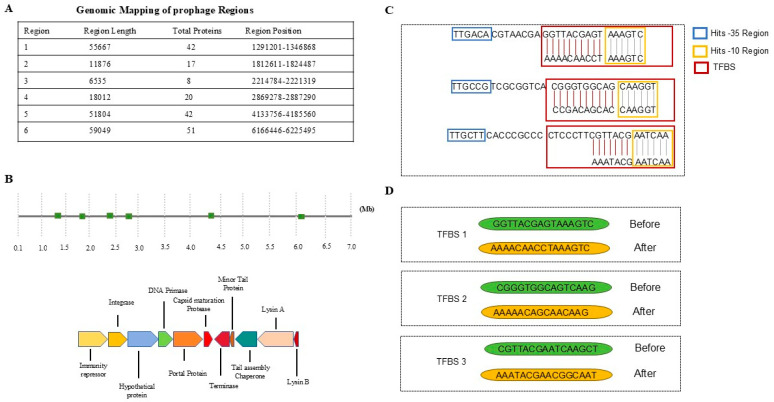
Identification and Activation of Prophages. (**A**) The genomic analysis identified six prophage regions, with the first prophage region chosen for further investigation. (**B**) A genome map is presented, annotated with the identified prophage regions. (**C**) Promoter motifs, including the -35 region (depicted in blue), the -10 region (depicted in yellow), and TFBS (depicted in red) are mapped. (**D**) The sequences of the TFBS are illustrated both before and after modifications aimed at reducing repressor binding affinity.

Following the initial screening, the platform meticulously conducts detailed prophage characterization, integrating multiple layers of genomic analysis to dissect the structural and functional architecture of each candidate prophage. Utilizing an advanced sequence parsing algorithm, the system effectively identifies key phage elements, including integrases, recombinases, structural proteins, and genes associated with lysis. This capability facilitates a robust differentiation between complete, inducible prophages and fragmented or cryptic elements. To validate prophage identity and classify them into potential families or clusters, comparative homology searches are conducted against curated viral databases. The analytical pipeline further investigates synteny and modular gene organization, which are essential characteristics of temperate phages, to enhance boundary precision and provide evolutionary insights. This comprehensive approach ensures that each prophage region is not only detected but also thoroughly annotated and contextually interpreted within the broader framework of phage biology. This process involves the precise delineation of prophage boundaries and, crucially, the identification of attachment sites, such as attL and attR. This step is crucial, as the recombination junctions determine the integration of the prophage into the host chromosome and its subsequent excision, rendering their precise localization vital for elucidating the molecular mechanisms that regulate prophage latency and activation. Our examination of the Bxb1 prophage (AF271693.1) in *Mycobacterium smegmatis* exemplifies this process (Figure 1): the platform accurately identified its site-specific integration at the attB site within the groEL1 gene, precisely mapping the 51,804 base pair prophage and its distinct attL (1494–1510 bp) and attR (39,808–39,824 bp) junctions. In addition to structural mapping, the platform is designed to facilitate comprehensive gene annotation within the prophage region, enabling the prediction of protein-coding sequences and the inference of potential functional roles throughout the phage life cycle.

A fundamental aspect of the platform’s innovative design is its emphasis on the deconstruction and engineering of regulatory circuits. The platform employs computational techniques to screen, annotate, and map a comprehensive array of regulatory elements within identified prophages, including promoter regions, TFBS, and other cis-regulatory modules. These elements collectively govern the precise temporal and spatial expression of phage genes. By integrating multiple algorithms, such as PWM-based searches and comparative genomics against experimentally validated regulatory motifs, the platform provides a detailed mapping that not only enables accurate predictions of promoter strength and regulatory hierarchy but also facilitates the identification of phage-encoded repressors and activators. These elements play a crucial role in modulating the switch between lysogenic and lytic developmental pathways.

The platform is designed to identify motifs and predict their functional roles in modulating gene expression throughout various stages of prophage-host interactions, with a particular emphasis on conserved bacterial RNA polymerase recognition sites such as the -35 and -10 regions. This comprehensive understanding of regulatory networks is crucial. The results have been corroborated by prior experimental studies [30,31,32]. In contrast to conventional prophage prediction tools, which typically concentrate on structural elements and often overlook regulatory insights, our platform uniquely combines promoter architecture analysis, TFBS mapping, and functional annotation into an integrated pipeline. For instance, while tools such as PHASTER and Prophage Hunter are capable of identifying prophage boundaries, they cannot provide detailed predictions of regulatory motifs or facilitate theoretical editing of promoters and repressor elements. This comparative advantage enables our platform not only to annotate latent prophages but also to inform rational design strategies for phage activation, representing a significant advancement in the field of computational phage therapy development. Ultimately, the platform is designed to facilitate targeted prophage modulation. By identifying and allowing for the theoretical manipulation of key TFBS, it serves as a powerful computational tool to investigate how specific nucleotide alterations can modify regulatory control to activate therapeutic phages in AMR bacteria.

### 3.2. A Universal Platform for Prophage Characterization and Modulation in Diverse Bacterial Lineages

**Figure 2:**
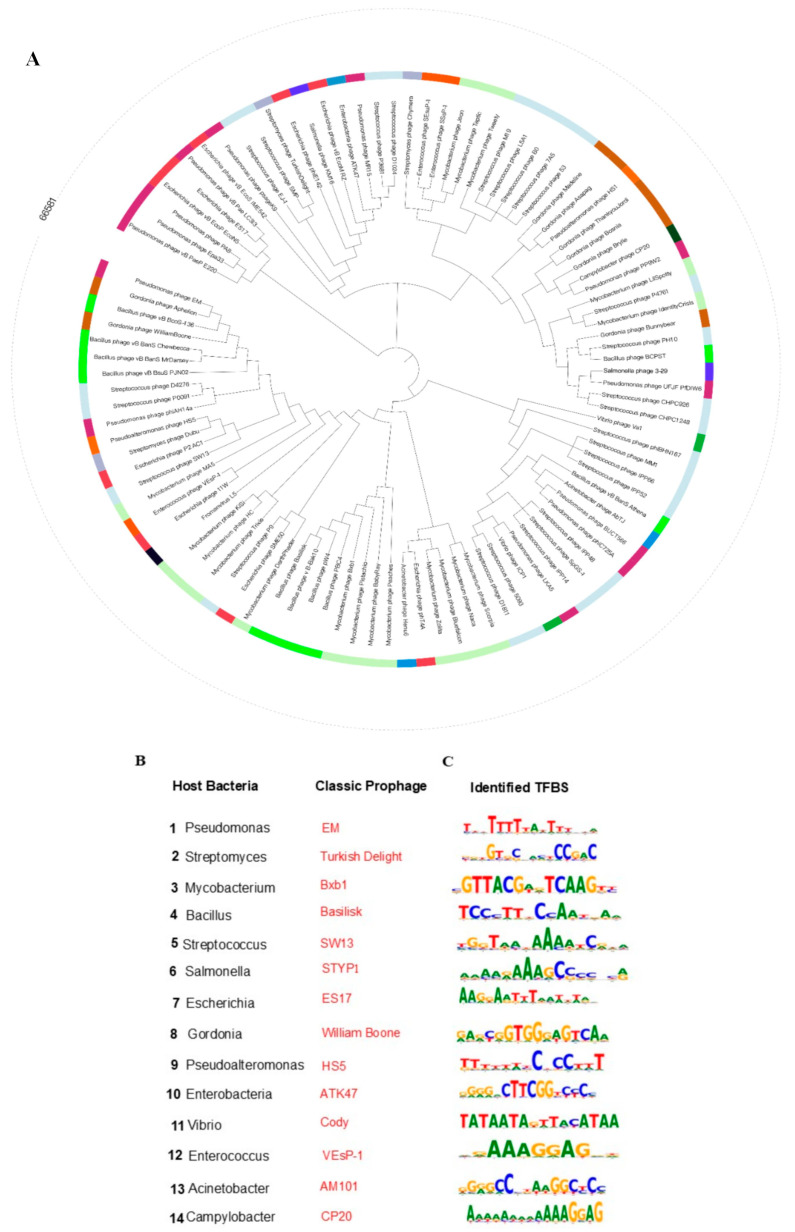
(**A**) A circular phylogenetic tree illustrating various host bacteria, with each genus distinctly color-coded for clarity. (**B**) Software detected prophages associated with these hosts and (**C**) their corresponding original TFBS sequences.

To evaluate the phylogenetic diversity of prophages across various bacterial lineages, we acquired complete genome sequences from a wide range of bacterial species, including representative genera such as *Bacillus*, *Streptococcus*, *Pseudomonas*, *Salmonella*, *Escherichia, Gordonia*, *Streptomyces*, and *Mycobacterium*. Employing our prophage activation platform, we systematically analyzed these genomes and successfully identified integrated prophages. The phylogenetic analysis encompassed a diverse array of bacterial hosts and their corresponding prophages (Figure 2A). This analysis effectively elucidated the evolutionary trajectories of prophages across different bacterial lineages, underscoring the platform’s capability for comprehensive characterization (Figure 2B). The platform’s reliability and precision in analyzing bacterial genomes and their associated prophages are demonstrated by its systematic identification and mapping of prophage regions, including attL and attR attachment sites.

These findings illustrate the capability of our prophage activation platform to analyze datasets of prophages associated with AMR bacteria, thereby identifying TFBS for modulating phage-host interactions. Our platform uniquely integrates two complementary prophage activation techniques to harness these elements for therapeutic development. Initially, we employed computational TFBS prediction algorithms to systematically identify conserved regulatory motifs within prophage genomes. Subsequently, to engineer the conditional activation of prophages, we utilized prophage activation-mediated editing to introduce activatable TFBS variants. The “Original TFBS” column displays the natural regulatory motifs identified, while the “Changed TFBS” column demonstrates the tool’s ability to modify these sequences (Figure 2C and Supplementary Table S2). Through the engineering of alterations in TFBS, our platform facilitates precise regulation of prophage gene circuits via the activation of prophage promoters. This capability is particularly pertinent in dynamic environments, such as during antibiotic exposure or under host immune pressure, where resistance phenotypes are stringently regulated at the transcriptional level. These findings are consistent with recent studies indicating that even minor nucleotide variations in TFBS can substantially influence the expression of resistance genes and contribute to phenotypic heterogeneity within bacterial populations [33]. By providing a controlled framework for investigating these modifications, our platform offers novel opportunities to explore therapeutic strategies aimed at reversing ‘prophage repressor immunity,’ thereby enhancing phage-mediated bacterial eradication.

### 3.3. A Systematic, Open, and User-Friendly Phage Research Tool

**Figure 3:**
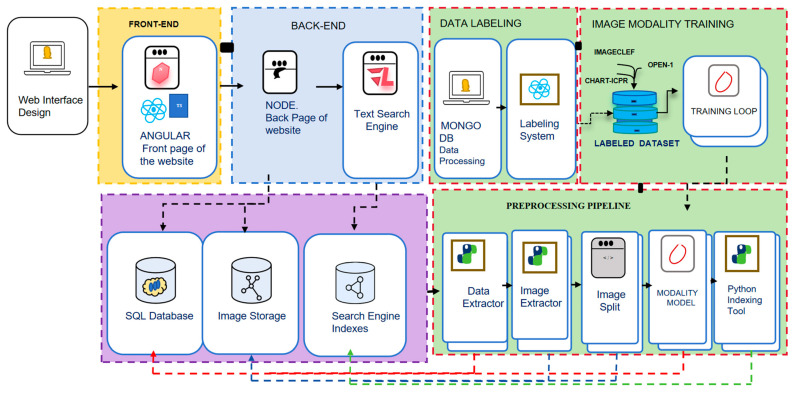
The system architecture of the web interface. The front-end is developed using Angular and TypeScript, while the back-end is constructed with Node.js and Apache Lucene. Data labeling is facilitated by MongoDB and a custom labeling system. The training of image modalities involves processing labeled datasets through a training loop. Data storage and management are handled using PostgreSQL, image storage solutions, and Lucene indexes. The preprocessing pipeline includes metadata parsing, image extraction, image splitting, modality modeling, and indexing with PyLucene. The entire workflow is initiated by a researcher activating prophages, inputting data into the system, and employing the taxonomy of modalities for comprehensive analysis.

The prophage activation platform is a standalone, interactive web application designed for local deployment via Docker [34]. The frontend is developed using Angular (https://angular.io) v5 [35] and TypeScript, providing a responsive interface accessible through web browsers. It leverages RxJS for asynchronous task management and Angular Material to ensure consistent UI components. The backend is implemented with Node.js (https://nodejs.cn/) v18.17.0 [36] and Python 3.12.4 (https://www.python.org/) v3.12.4 [37]. For persistent data storage, the application utilizes MongoDB (https://www.mongodb.com/) v6.0 [38], integrated through Mongoose v7.6 to enable efficient schema management [39]. Genomic and omics data processing is conducted using scikit-learn v1.3.2 for machine learning applications, while Seaborn v0.13.2 and Pyplot v3.8.2 are employed for data visualization [40]. The overall system architecture of the web platform is presented in Figure 3. The platform’s scalability in handling large datasets is enhanced through the use of sparse matrices and multithreading. The platform has undergone extensive testing on Unix-based systems, including Linux and macOS, as well as on Windows [41,42]. Within the Prophage Activation platform, users are mandated to employ nucleotide sequence files in FASTA format at every stage of the analysis (Supplementary Figure S5). During the second step, which entails the identification of the attL and attR attachment sites, users are required to upload a corresponding GenBank file (.gbk), as it provides critical annotation data necessary for the accurate delineation of the prophage region boundaries. Subsequent stages permit users to upload sequence files to identify promoter regions, investigate TFBS, and assess promoter activity while eliminating repressor elements. The interface for each stage ensures that users submit data in a standardized format, thereby facilitating seamless processing and ensuring precise analytical outcomes.

### 3.4. Comprehensive Workflow of the Prophage Activation Platform Across Diverse Bacterial Hosts

**Figure 4:**
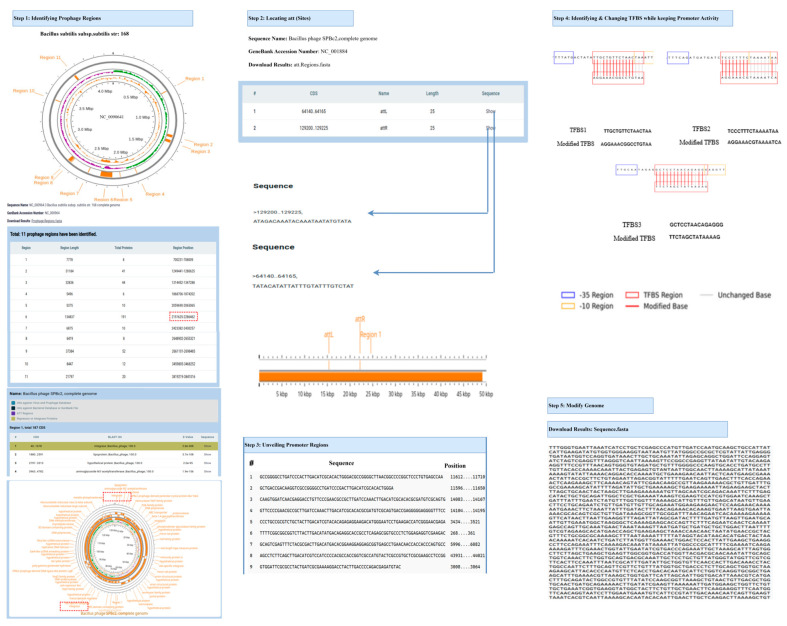
Detailed, comprehensive workflow of the Prophage activation of *Bacillus subtilis* subsp.subtlis str.168.

Figure 4 and Supplementary Figures S6 and S7 comprehensively depict the detailed workflow of the Prophage Activation Platform as applied to the genomes of three distinct bacterial hosts: *Bacillus subtilis*, *Pseudomonas aeruginosa*, and *Gordonia terrae*, along with their respective prophages. The workflow initiates with the selection and uploading of a complete bacterial genome, which is subsequently subjected to genome-wide prophage detection. This process identifies multiple candidate prophage regions, detailing their lengths, positions, and protein content. Each identified prophage region is then associated with a known phage genome through homology analysis, followed by the annotation of the phage genome to identify protein-coding genes and functional modules. Finally, site-specific recombination regions, namely attachment sites (attL and attR), are extracted from both bacterial and phage sequences utilizing precise positional information. These integration junctions offer valuable insights into phage-host interactions. Subsequently, the platform conducts promoter region mapping, pinpointing conserved bacterial promoter motifs—particularly the -35 and -10 elements—and determining their locations throughout the prophage genome. TFBS are then identified and visualized within or adjacent to these promoters. The workflow allows for precise modifications of these regulatory elements by altering the TFBS while maintaining the core activity of the promoter, thereby simulating enhanced or suppressed gene expression potential. Ultimately, the modified prophage genome is reconstructed and exported as an engineered DNA sequence, suitable for subsequent experimental design or synthetic biology applications. This comprehensive approach facilitates the rational reprogramming of prophage regulatory elements, thereby enabling functional studies and the regulation of activation in a variety of bacterial hosts.

### 3.5. Comparative Performance Assessment of Prophage Prediction Tools

**Figure 5:**
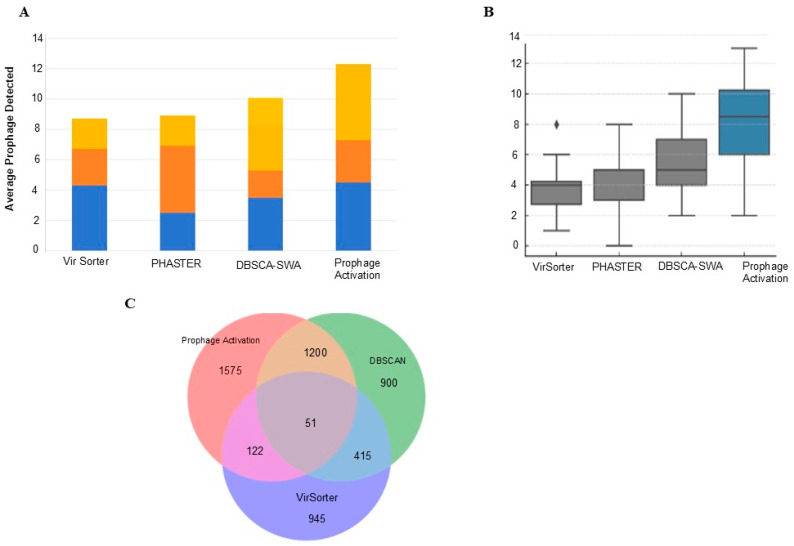
Comparative Performance Assessment of Prophage Activation Platform, PHASTER, VirSorter, and DBSCAN-SWA. (**A**). Average number of prophages detected per genome by different tools, colors indicate stacked counts across genomes. Prophage_Activation consistently identifies more prophages than other tools (**B**). Distribution of prophages detected per genome across tools (symbols represent boxplot elements: median line, quartiles as boxes, whiskers for range, and dots for outliers) (**C**). Shared and unique prophages identified by Prophage activation, DBSCAN, and Vir Sorter.

To systematically assess the performance of the Prophage Activation Platform, we compared its predictions with those of widely used prophage detection tools, including PHASTER, VirSorter, and DBSCAN-SWA, across a diverse dataset of bacterial genomes (Supplementary Table S4). On average, our platform identified a greater number of prophage regions per genome than the other tools, with consistent performance across multiple bacterial taxa (Figure 5A). In terms of prophage distribution, the Prophage Activation Platform demonstrated broader detection sensitivity, capturing smaller or fragmented elements frequently missed by alternative approaches (Figure 5B). Comparative overlap analysis further revealed that while a substantial number of prophages were shared among tools, the Prophage Activation Platform identified 1575 unique prophage regions not detected by DBSCAN-SWA or VirSorter, highlighting its enhanced sensitivity and ability to capture regulatory features overlooked by other methods (Figure 5C).

In addition to boundary identification, our platform uniquely integrates regulatory annotation by systematically identifying promoters and TFBS within prophage regions. This regulatory layer, absent from existing prophage detection tools, provides insights into the functional potential of predicted prophages. Together, these results underscore that the Prophage Activation Platform not only performs competitively in prophage detection but also offers a complementary dimension of regulatory analysis that broadens its applicability to synthetic biology and therapeutic phage design.

## 4. Discussion

Traditional phage therapy is hindered by notable limitations, such as the necessity for ongoing phage discovery and constraints related to host specificity. In contrast, our approach emphasizes the precise identification and modification of TFBS, which facilitates the therapeutic phage’s escape from prophage repressors, thereby circumventing the challenges associated with isolating new therapeutic phages. The outcomes derived from our Prophage Activation Platform underscore the feasibility and robustness of this methodology. Our bioinformatics pipeline effectively identified and analyzed TFBS within dormant prophages, uncovering critical regulatory sequences that sustain lysogeny. By strategically modifying these sequences while maintaining essential promoter functions, we computationally predicted repressive mechanisms and induced lytic activation.

A significant advancement presented by our Prophage Activation Platform is its ability to transcend the static detection of prophage elements, a methodology predominantly employed by existing tools such as PhiSpy, Prophage Hunter, PHASTER, DBSCAN, Seeker, and Phage Finder. While these tools have been instrumental in the initial phases of prophage discovery, they are limited to boundary identification based on heuristic rules, sequence composition, or similarity searches. For example, PhiSpy and Seeker utilize gene content and compositional bias to delineate prophage regions, whereas PHASTER and Prophage Hunter combine homology searches with viral gene cluster analysis to pinpoint potential prophage loci. PHATEST provides a user-friendly web interface that facilitates prophage analysis in large bacterial genomes, making it a widely accessible resource. In contrast, our Prophage Activation Platform is intended as a complementary resource, focusing on regulatory elements such as TFBS and promoter analysis, which are not addressed by PHATEST”. DBSCAN employs density-based spatial clustering to identify genomic islands, and Phage Finder emphasizes similarity to known phage genes.

However, none of these platforms evaluate the regulatory networks embedded within the identified prophages, nor do they provide tools for manipulating lysogeny-associated genetic switches. In comparative analysis, our Prophage Activation Platform demonstrated enhanced sensitivity relative to widely used prophage detection tools. Rather than exceeding existing platforms, our approach is designed to be complementary, providing additional regulatory insights specifically TFBS and promoter analysis, which are not currently addressed by most prophage detection tools.

Our platform addresses these limitations through a sophisticated multilayered architecture that integrates high-resolution prophage identification with predictive modeling of regulatory elements crucial for prophage control. Specifically, we incorporate a motif-aware recombination module that precisely localizes attL and attR sites, which are critical boundaries frequently overlooked or misannotated by other tools. These recombination junctions, especially when located near integrase genes, are vital for elucidating integration specificity and lytic inducibility. Additionally, while existing tools typically only annotate genes or identify repressor-encoding open reading frames (ORFs), our platform goes further by analyzing the functional binding landscape of these repressors. This is achieved by identifying associated TFBS through both curated database matching and de novo motif discovery. Consequently, our platform enables the reconstruction of the transcriptional logic governing prophage activity, a capability not present in any existing prophage tools.

Unlike existing platforms that do not possess promoter recognition capabilities, our pipeline employs machine learning-based promoter prediction, trained on a substantial dataset of experimentally validated bacterial promoters. This approach enables the precise identification of core-10 and -35 boxes, the assessment of spacing rules, and the integration of promoter information into the TFBS scanning module. In contrast, tools such as PHASTER, Prophage Hunter, and Seeker cannot assess the regulatory potential of intergenic regions or quantify the binding affinity of regulatory proteins, thus constraining their effectiveness in designing functional interventions.

Our platform uniquely facilitates the targeted re-engineering of TFBS, a capability that distinguishes our system. By employing a bespoke scoring framework that evaluates binding site strength, positional entropy, and cross-species conservation, we enable rational editing of TFBS. This allows users to diminish repressor affinity while maintaining promoter functionality, a sophisticated design feature crucial for the precise engineering of therapeutic phages. Currently, no existing tool offers such predictive, simulation-based editing of cis-regulatory elements. For instance, PropagAtE attempts to estimate prophage activity through metagenomic read coverage ratios but does not interact with the regulatory DNA sequence itself, nor can it model the effects of specific mutations at the transcriptional control level.

Moreover, our user interface and output pipeline distinctly set our platform apart from existing solutions. While PHATEST and Prophage Hunter provide web access, their functionalities are confined to static visualizations and summary tables. In contrast, our web-based platform not only facilitates high-throughput processing and multi-genome comparisons but also offers an interactive visualization environment. This environment enables users to examine the att site architecture, promoter maps, TFBS motifs, and repressor-target relationships. This design is particularly advantageous for users engaged in synthetic biology applications, such as engineering inducible prophages for phage therapy.

Importantly, while our pipeline predicts regulatory elements that may be targeted for prophage activation, to our knowledge, there are no documented clinical cases in which pathogenic bacteria have been eradicated solely through deliberate prophage activation. Laboratory studies have shown that prophages can be induced by stressors such as UV irradiation, antibiotic exposure, or DNA damage, but these findings have not yet translated into therapeutic applications. This underscores that our results represent computational hypotheses and highlights the need for experimental validation. Nevertheless, prophages naturally tend to revert to dormancy, and achieving stable activation remains a significant challenge. Stable lytic bias may therefore require multiplex genome edits, such as combining repressor/operator disruption with integrase modulation. This will be a priority for future work and experimental validation. Prophage elements are not isolated units but are embedded within bacterial chromosomes, where their interactions with neighboring genes and regulatory networks can influence host fitness, pathogenicity, and responsiveness to induction. Highlighting this genomic integration underscores the biological complexity of prophages and further justifies the need for computational tools, such as ours, that can systematically map and analyze their placement within host chromosomes. We hope these biological relevance of our study will align our work more closely with practical applications in phage biology and its application on therapy in the future.

Despite these contributions, several limitations of our study should be acknowledged. First, the current work is entirely computational and does not include wet-lab validation of predicted targets. Second, our dataset was restricted primarily to Actinobacteriophages, which limits the scope of generalization. Future benchmarking across a broader range of bacterial taxa, including *Proteobacteria*, *Firmicutes*, and other clinically relevant groups, will be necessary to comprehensively validate the platform. Third, while BLAST searches with an E-value cutoff of ≤10^−7^ were effective for prophage and attachment site detection, more sensitive approaches such as Hidden Markov Models (HMMs) may be required to detect highly divergent homologs. Finally, although we applied the pipeline to ~90 genomes, demonstrating its scalability, large-scale benchmarking across hundreds or thousands of genomes will be essential for robust validation. Future work will therefore focus on expanding dataset diversity, refining motif detection strategies, and collaborating with experimental laboratories to test these predictions in vitro and in vivo.

Ultimately, our platform represents an unparalleled online resource that empowers researchers to thoroughly investigate prophage–host interactions and the regulatory architecture associated with AMR genes. The integration of large-scale data analysis with precise editing of regulatory elements provides a foundation for developing novel therapeutic phage strategies against AMR.

## Data Availability

Data is contained within the article or Supplementary Material.

## Supplementary Materials

The following supporting information can be downloaded at: https://www.mdpi.com/article/10.3390/life15091417/s1, Figure S1: The overview of the Prophage Activation Web Server; Figure S2: Identification of Prophage Regions and Attachment Sites; Figure S3: Pinpointing Promoter Regions; Figure S4: Pipeline for Locating and Changing TFBS and Scoring System; Figure S5: A structured workflow is used for prophage Activation; Figure S6: Detailed, comprehensive workflow of the Prophage activation of *Pseudomonas aeruginosa* strain PAK; Figure S7: Detailed, comprehensive workflow of the Prophage activation of *Gordonia terrae* strain 3612 Chromosome; Table S1: Prophage Activation and Lytic Conversion: Predicted Prophages and Related Information in Different Phage Genomes Across Diverse Bacterial Species; Table S2: Comparative Analysis of Existing Prophage Detection Tools and Their Functional Capabilities; Table S3: Prophage Activation Across Diverse Bacterial Species Using Training Organisms; Table S4: Comparative analysis of Prophage Activation with other prophage detection tools; Table S5: Benchmarking of Prophage Identification and Activation Tools Across Functional and Performance Dimensions; The computer source code is available from the public GitHub repository: https://github.com/Lou-lab-01/Prophage_Activation (accessed on 5 May 2025).

## Abbreviations

The following abbreviations are used in this manuscript:

AMR	Antimicrobial Resistance
MCC	Matthews Correlation Coefficient
NCBI	National Center for Biotechnology Institute
ORF	Open Reading Frame
PWM	Position Weight Matrix
RF	Random Forest
SIE	Super Infection Exclusion
TFBS	Transcription Factor Binding Site
