# Supplementary material for: Prophage Activation: An In Silico Platform for Identifying Prophage Regulatory Elements to Inform Phage Engineering Against Drug-Resistant Bacteria
Source: Life (Basel). 2025 Sep 8;15(9):1417. doi: 10.3390/life15091417 (PMC12471524; doi:10.3390/life15091417)
Supplement: Supplementary file 1 [file life-15-01417-s001.zip › Supplementary Figures.pdf]

Supplementary Figures

Prophage Activation: An In-Silico Platform for Identifying Prophage Regulatory Elements to Inform Phage Engineering Against Drug-Resistant Bacteria

Saher Musrrat [1], Zequan Han [2], Kai Wang [3], Yunhai Huang [3], Yanhui Xiang [1,*], Sen Liu [2,*], and Wen Yin [1,*]

[1] Center for Cell and Gene Circuit Design, CAS Key Laboratory of Synthetic Genomics, Shenzhen Institute of Synthetic Biology, Shenzhen Institutes of Advanced Technology, Chinese Academy of Sciences, Shenzhen 518055, China

[2] Hubei University of Technology, Wuhan 430068, China

[3] State Key Laboratory of Bioreactor Engineering and School of Biotechnology, East China University of Science and Technology, Shanghai 200237, China

* Authors to whom correspondence should be addressed.

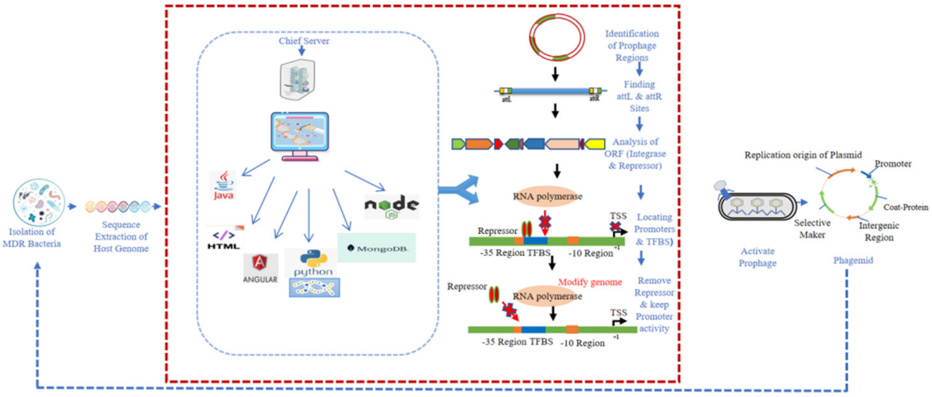

Supplementary Figure S1. The overview of the Prophage Activation Web Server.

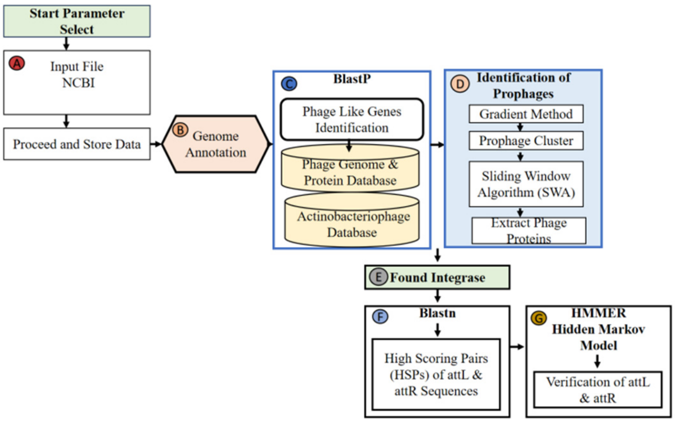

Supplementary Figure S2. Identification of Prophage Regions and Attachment Sites.

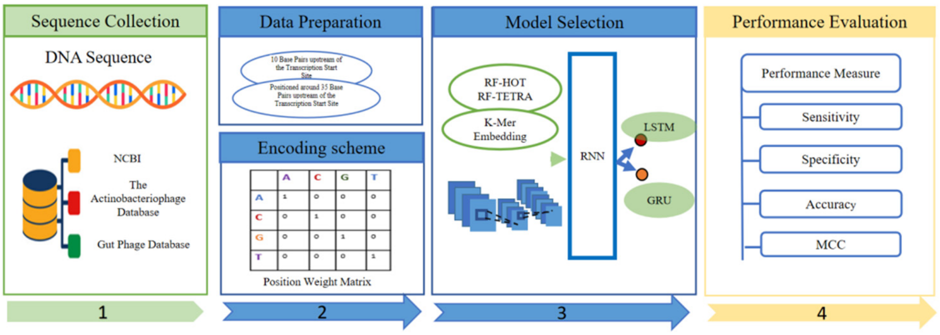

Supplementary Figure S3. Pinpointing Promoter Regions.

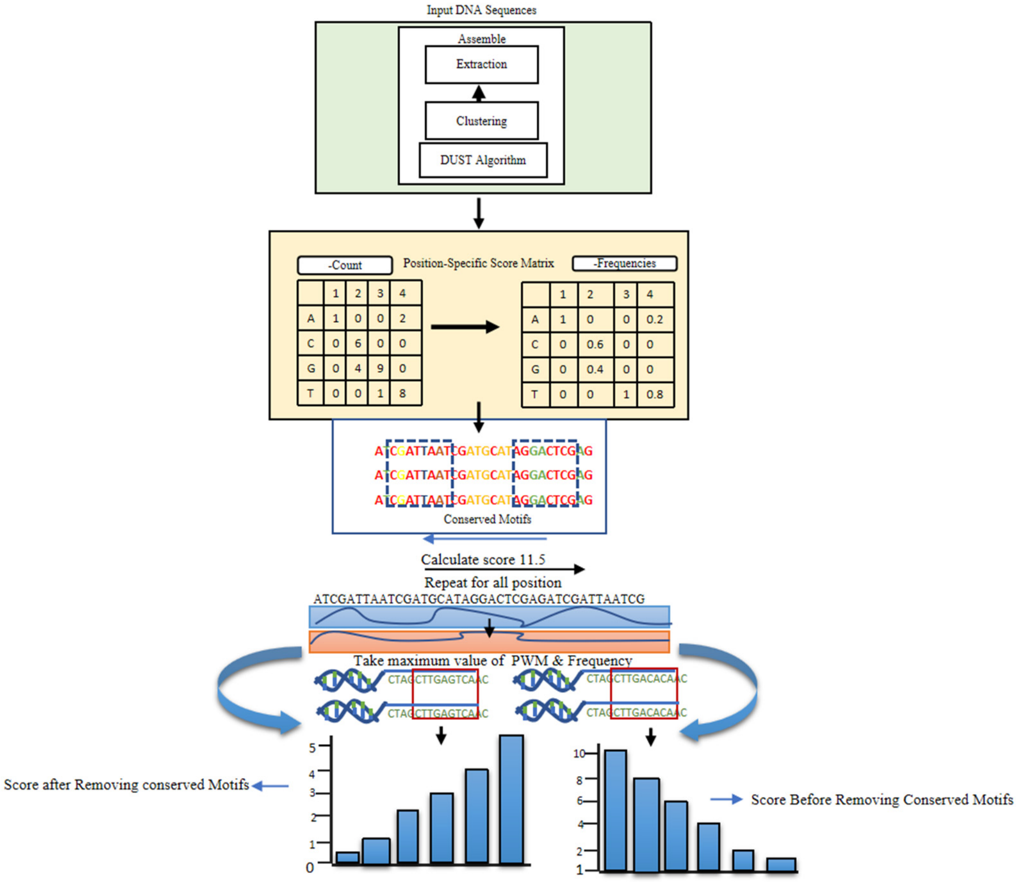

Supplementary Figure S4. Pipeline for Locating and Changing TFBS and Scoring System.

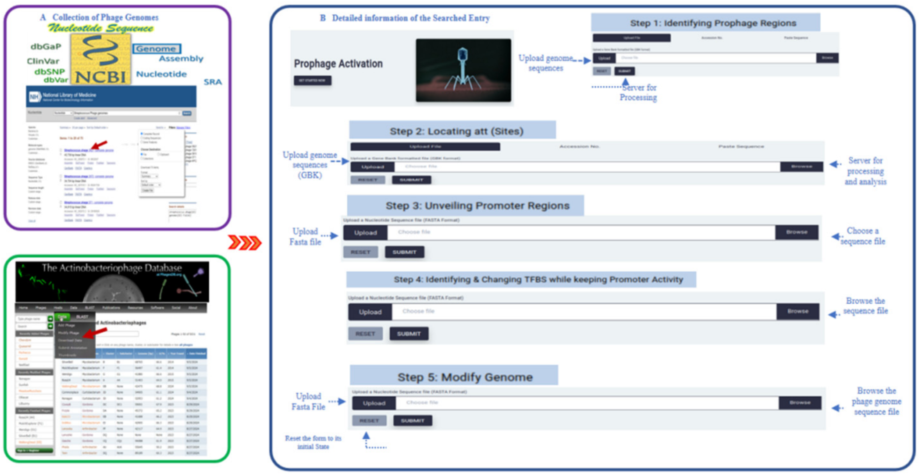

Supplementary Figure S5. A structured workflow is used for prophage Activation.

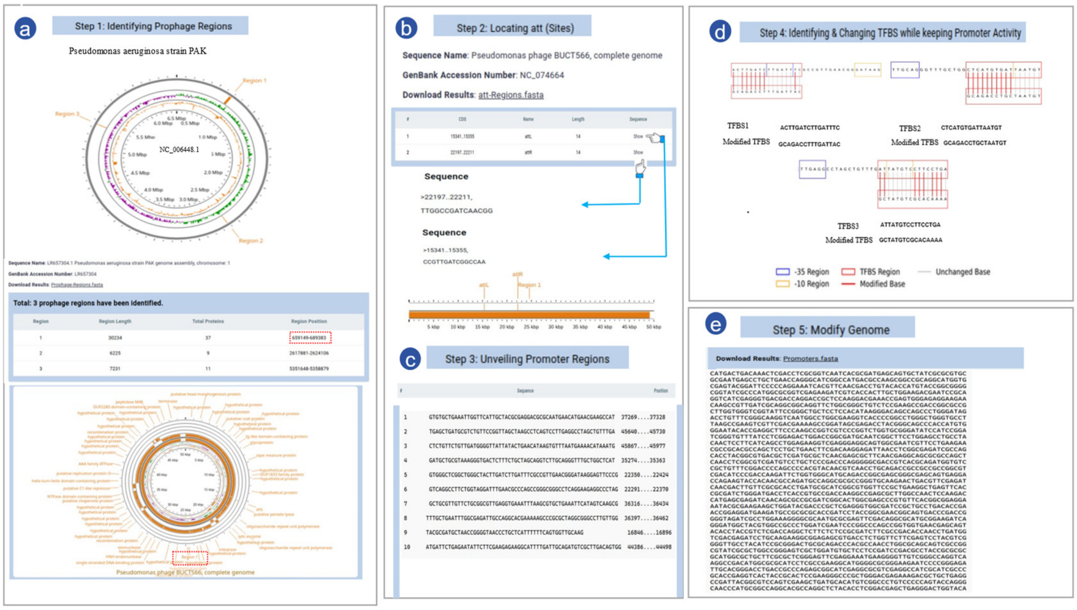

Supplementary Figure S6. Detailed, comprehensive workflow of the Prophage activation of Pseudomonas aeruginosa strain PAK.

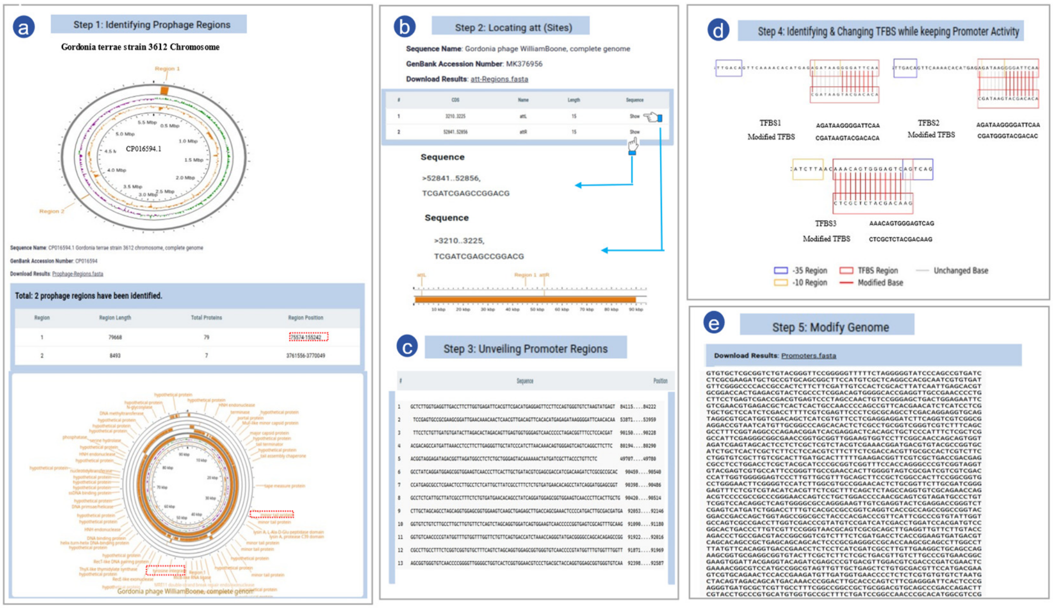

Supplementary Figure S7. Detailed, comprehensive workflow of the Prophage activation of Gordonia terrae strain 3612 Chromosome.
